# Prevalence and factors associated with diabetic ketoacidosis at diagnosis of type 1 diabetes: A report from a tertiary medical center in Central Pennsylvania

**DOI:** 10.1002/edm2.186

**Published:** 2020-09-12

**Authors:** Kaleb Tadesse Bogale, Daniel E. Hale, Eric Schaefer, Kanthi Bangalore Krishna

**Affiliations:** ^1^ Penn State College of Medicine Hershey PA USA; ^2^ Division of Pediatric Diabetes and Endocrinology Penn State Hershey Medical Center Hershey PA USA; ^3^ Department of Public Health Sciences Penn State Hershey Medical Center Hershey PA USA

**Keywords:** affordable care act, diabetic ketoacidosis, type 1 diabetes

## Abstract

**Objective:**

To explore the rate and factors associated with diabetic ketoacidosis (DKA) at diagnosis of type 1 diabetes (T1D) in a single tertiary medical centre in Central Pennsylvania.

**Methods:**

Retrospective chart review of all individuals ≤ 18 years of age who were diagnosed with T1D (N = 350) at the Penn State Hershey Pediatric Diabetes Clinic from January 2017 to December 2019. We report logistic regression models for DKA at diagnosis of T1D for age, gender, race/ethnicity, BMI percentile, health insurance, outcome of any healthcare encounter 30 days prior to T1D diagnosis, HbA_1_c level, altered mental status at diagnosis, and diagnosis of autism spectrum disorder and a multivariable logistic regression model including all aforementioned variables.

**Results:**

Of the 350 newly diagnosed children with T1D from 2017 to 2019, 161/350 (46%) presented in DKA. Among patients with DKA, there were 45 (28%) in mild DKA and 116 (72%) in moderate/severe DKA, which represents 13% and 33% of all patients diagnosed with T1D, respectively. Variables associated with increased risk of DKA at presentation of T1D included age (<3 or 9‐13), BMI percentile (<3% or > 97%), no referral during preceding healthcare encounter, HbA_1c_ level and altered mental status. In a multivariable model, age (<3 or 9‐13), no referral during preceding healthcare encounter, HbA_1_c level and altered mental status were associated with DKA at presentation, whereas gender, race/ethnicity, BMI percentile, health insurance and autism spectrum disorder diagnosis were not.

**Discussion:**

Our study notes an overall higher rate of DKA at diagnosis (46%) compared to the SEARCH study (approximately 30%) but a lower rate compared to a recent study in Colorado children (58%).

## Graphical abstract

To explore the rate and factors associated with diabetic ketoacidosis (DKA) at diagnosis of type 1 diabetes (T1D) in a single tertiary medical centre in central Pennsylvania, we report a retrospective chart review of all individuals < 18 years of age who were diagnosed with T1D (N = 350) at the Penn State Hershey Pediatric Diabetes Clinic from January 2017 to December 2019. Among the 350 newly diagnosed children with T1D, there were 45 (13%) in mild DKA and 116 (33%) in moderate/severe DKA. In a multivariable model, age (<3 or 9‐13), no referral during preceding healthcare encounter within 30 days, HbA1c level, and altered mental status were associated with DKA at presentation, whereas gender, race/ethnicity, BMI percentile, health insurance and autism spectrum disorder diagnosis were not.

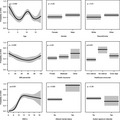

## 1 INTRODUCTION

Diabetic ketoacidosis (DKA) at the time of diagnosis in type 1 diabetes (T1D) is a preventable complication given the recognizable symptoms (polyuria, polydipsia, enuresis, weight loss and fatigue) of T1D.^1^, ^2^ Children diagnosed in DKA have increased morbidity, mortality, and incur higher medical costs and healthcare resource utilization including ICU level care.^3^ Several previous studies have identified risk factors for children to present in DKA, including age (<5 or 10‐12),^4^ Hispanic and African American race, low socioeconomic status, misdiagnosis at an initial encounter and lack of private health insurance.^5^, ^6^, ^7^, ^8^ A recent study in Colorado children was the first to report similar rates of DKA among privately insured children and Medicaid aligning with the implementation of the Affordable Care Act (ACA) in 2010.^9^ Established rates of DKA at diagnosis of T1D differ between different countries and states within the United States.^8^, ^10^ The aims of this study were to (a) determine the rate of DKA at presentation of T1D at a single tertiary medical centre in central Pennsylvania and (b) evaluate the factors associated with their presentation in DKA.

## 2 METHODS

### 2.1 Research design and methods

Retrospective chart review of all individuals ≤ 18 years of age who were diagnosed with T1D at the Penn State Hershey Pediatric Diabetes Clinic from January 2017 to December 2019. All clinical documents, including scanned media from outside hospitals, were manually reviewed for the 30 days leading up to the initial diagnosis of T1D. The medical record was abstracted 6 months following onset of T1D to confirm the diagnosis. All patients included in the study had at least one of three autoantibodies positive (Islet Cell Cytoplasmic Autoantibodies [ICA], Insulin Autoantibodies [IAA] and Glutamic Acid Decarboxylase Autoantibodies [GADA]). Children with ‘other’ forms of diabetes including cystic fibrosis‐related diabetes, type 2 DM, medication‐induced diabetes or other causes of metabolic acidosis were excluded. De‐identified data extracted for every patient includes age, gender, race/ethnicity, BMI percentile, health insurance, outcome of any healthcare encounter (primary care provider or specialist) 30 days prior to diagnosis of T1D, autism spectrum disorder diagnosis, family structure, family history of T1D, symptoms at diagnosis noted in medical documentation, and laboratory values at diagnosis (blood glucose, HbA_1c_, venous pH). The pH at diagnosis was used to classify DKA as mild (7.25‐<7.3) or moderate/severe (<7.25).

### 2.2 Statistical analyses

First, differences in diagnoses were examined by each variable. Chi‐squared tests were used to test for differences in rates of DKA at diagnosis of T1D for the following variables: age at diagnosis, gender, race/ethnicity, BMI percentile, health insurance, outcome of any preceding healthcare encounter 30 days prior to diagnosis of T1D (immediate referral, no referral, emergency department [ED]), HbA_1c_, altered mental status and diagnosis of autism spectrum disorder. A logistic regression model for DKA diagnosis was generated separately for each variable. The fitted logistic regression models were shown graphically using estimated probabilities. A multivariable logistic regression model was fit for all variables aforementioned. Odds ratios (ORs) and corresponding 95% confidence intervals (CIs) were reported from the models. A total of 16 patients (5%) had a missing value for at least one of the variables included in the multivariable model and were subsequently excluded when fitting the model.

## 3 RESULTS

### 3.1 Patient population

There were 350 patients aged < 1 to 18 included in the study. The peak occurrences of T1D were in ages 7‐13 (IQR), males (205, 59%) and white race (249, 71%) with a subset of 12 (3%) Amish children. The majority of children had private insurance (190, 54%) or Medicaid (133, 38%) coverage. There were 21 (6%) uninsured children, 4 (1%) with military/government insurance and 2 (%1) lacked information. There were 25 (7%) children with autism spectrum disorder and 33 (10%) with attention deficit hyperactivity disorder. There was a bimodal distribution for BMI percentile, with 54 patients (15%) <15th percentile and 43 patients (12%) >85th percentile. There were 225 (64%) children with parents that were married, 53 (15%) single parents, 31 (9%) divorced parents, 32 (9%) legal guardians and 9 (3%) lacked information. The majority (330, 94%) of children spoke English as their primary language and 20 (6%) spoke English as a second language. The majority (326, 93%) of patients reported polyuria, polydipsia, or nocturia, 199 (58%) reported weight loss, and 20 (5.7%) reported altered mental status.

### 3.2 Rate of DKA

Of the 350 newly diagnosed children with type 1 diabetes from 2017 to 2019, 161/350 (46%) presented in DKA (95% CI: 41%‐51%). Among patients with DKA, there were 45 (28%) in mild DKA and 116 (72%) in moderate/severe DKA, which represents 13% and 33% of all patients diagnosed with T1D, respectively.

### 3.3 Family history

The majority of children (216, 66%) reported no family history of T1D, 39 (12%) had a first degree relative, 71 (22%) had a second degree relative and 24 (7%) lacked information. Children with a first degree relative with T1D had the lowest rate of DKA at 28%, compared to children with a second degree relative (56%) or no family history (46%).

### 3.4 Pathway to diagnosis

Almost all patients (98%) had an established primary care provider at the time of diagnosis of T1D. The majority of patients (259, 74%) had not received care within Penn State Health, while 29 (8%) and 62 (18%) received primary or specialty care. All patients either had a healthcare encounter within 30 days of their diagnosis of T1D (250, 71%) or presented to the ED directly (90, 26%). Patients with any healthcare encounter within 30 days of their diagnosis of T1D were classified as either ‘immediate referral’ or ‘no referral’ group, depending if providers made a referral for concerns for T1D. In children with multiple healthcare encounters within 30 days of diagnosis, we only considered the outcome of the initial healthcare encounter. There were more children in the immediate referral group 203 (58%) than the no referral group 47 (13%). Children in the no referral group had the highest rate of DKA (75%), compared to the rates of DKA for children in the immediate referral group (37%) or those that presented to the ED directly (51%).

### 3.5 Clinical factors associated with DKA

**Figure 1:**
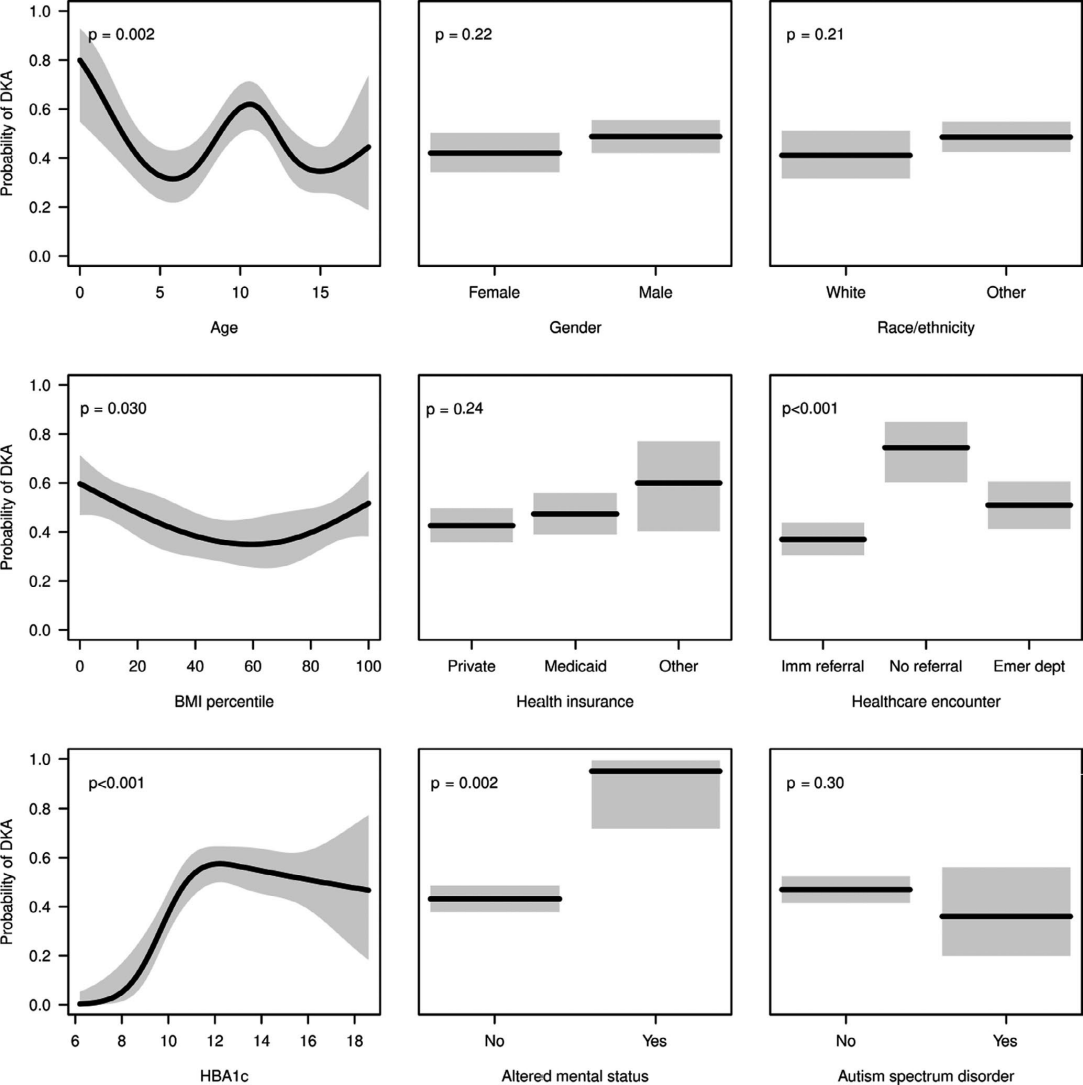
Estimated probabilities of DKA (black lines) and corresponding 95% CIs (grey regions) from logistic regression models fit separately for each variable

In univariable analysis, we found very young patients (<3) and patients aged 9 to 13 had the highest risk of DKA at diagnosis (Figure 1). Children at the extremes for BMI percentile (<15th or > 85th percentiles) had higher probability of being diagnosed with DKA. Children with a HbA_1_c ≥ 10% (86 mmol/mol) or altered mental status had a higher risk of DKA at diagnosis. There were no differences in DKA at diagnosis based on gender, ethnicity/race, autism spectrum disorder diagnosis or insurance coverage. Figure 1 shows the estimated probabilities of DKA from logistic regression models fit separately for each variable. In the multivariable model (Table 1), clinical factors associated with increased risk for presentation in DKA at diagnosis included age (<3 and 9‐13), no referral during any healthcare encounter 30 days prior to diagnosis, HbA_1_c ≥ 10% (86 mmol/mol) and altered mental status.

**Table 1:** Parameter estimates from multivariable logistic model of DKA at diagnosis of T1D

Parameter	OR (95% CI)	*P*‐value
Age		
0‐3	3.25 (1.16‐9.12)	**.025**
4‐8	1.19 (0.56‐2.53)	.65
9‐13	2.59 (1.36‐4.95)	**.004**
14‐18 (ref)	1	
Sex		
Male	1.52 (0.90‐2.56)	.12
Female (ref)	1	
Race		
White	1.78 (0.96‐3.29)	.07
Other (ref)	1	
BMI percentile		
<15th Percentile	1.35 (0.65‐2.79)	.42
15th to 85th Percentile	0.76 (0.41‐1.43)	.40
>85th Percentile (ref)	1	
Heath insurance		
Private (ref)	1	
Medicaid	1.11 (0.62‐1.98)	.73
Other	1.93 (0.74‐5.01)	.18
Healthcare encounter		
Immediate Referral (ref)	1	
No Referral	4.87 (2.07‐11.5)	**<.001**
Emergency Department	1.45 (0.82‐2.58)	.20
HbA_1c_		
≤10% (86 mmol/mol) (ref)	1	
>10% (86 mmol/mol)	6.94 (3.24‐14.9)	**<.001**
Altered mental status		
Yes	9.51 (1.15‐78.4)	**.036**
No (ref)	1	
Autism spectrum disorder		
Yes	0.89 (0.32‐2.43)	.82
No (ref)	1	

Bold values signify a *P*‐value < 0.05.

## 4 CONCLUSIONS

Our study was the first to report the rate of DKA at diagnosis of T1D within central Pennsylvania. From 2017 through 2019, our study had an average rate of DKA at presentation of T1D in children at 46%, of whom 72% were in moderate or severe DKA and 28% in mild DKA. Historically, the presentation of DKA at diagnosis of T1D in children has remained stable at approximately 30% in multiple geographic areas from 2002 through 2010 according to a United States‐based public health SEARCH study.^8^ The Pediatric Diabetes Consortium found an average rate of 34% presenting in DKA, half of whom presented with moderate or severe DKA, from 2009 through April 2011.^5^ A report from 2010 through 2013 compared rates of DKA at diagnosis of T1D with an earlier study in the same urban setting of Queens, New York 15 years earlier. There was a steady decrease of from 38% to 29% over this 15 year span, although it was unclear if this was due entirely to temporal differences.^7^

A recent report in Colorado children found the rate of DKA at diagnosis increased steadily from 2010 through 2017, reaching ~ 60% in 2017.^9^ They found no differences between children with private insurance and Medicaid insurance after the implementation of the ACA. While both Medicaid and privately insured children had an increasing rate of DKA from 2010 to 2017 in their study, there was a disproportionate increased rate of DKA among privately insured individuals. The authors proposed a potential driver is increased enrolment in high‐deductible private insurance plans that disincentive families from seeking timely care.^9^ We report similar findings of no difference in the risk of DKA at diagnosis based on private insurance or Medicaid in a patient population after the implementation of the ACA in 2010; however, we still note an overall lower rate of DKA than their study. Previous reports before the implementation or the first years of the ACA found children with private insurance had lower rates of DKA compared to Medicaid.^7^, ^11^, ^12^

An alarming proportion of children who had a healthcare encounter within 30 days of their diagnosis of T1D but who had not been referred for concern of T1D presented in DKA (75%), which was much higher than our average rate of DKA (46%), children in the immediate referral group (37%), or those that presented to the ED directly (51%). Our findings support previous reports that a misdiagnosis of a child presenting in T1D is associated with an increased rate of DKA.^7^, ^12^ One report found rates of misdiagnosis of T1D are highest among very young children (<5) and independent of type of healthcare insurance.^12^ Common misdiagnosis include gastroenteritis, respiratory infection, viral syndrome, yeast infection, and urinary tract infection.^7^ Our data suggest children without an immediate referral during a preceding healthcare encounter are at the greatest risk of presenting in DKA and reinforces the importance of appropriate diagnostic screening for T1D in the primary care setting. Limitations of this study include the nature of a retrospective study, which is limited by the accuracy of documented information provided within the medical record and all scanned media, and the single‐centre design.

In conclusion, our study was the first to report the rate of DKA at diagnosis of T1D within a tertiary medical centre in central Pennsylvania at 46% and the associated risk factors. Future interventions such as general public awareness campaigns, educating childhood contacts on the presenting symptoms of T1D or islet autoantibody screening for high‐risk populations were proposed to reduce the rate of children presenting in DKA at diagnosis of T1D.^13^, ^14^

## CONFLICT OF INTEREST

No authors report a conflict of interest.

## AUTHOR CONTRIBUTION

KTB and KBK performed the research. KTB, DH and KBK designed the research study. ES analysed the data. KTB, DH, ES and KBK wrote and edited the final manuscript.

## Data Availability

The data that support the findings of this study are available from the corresponding author upon reasonable request.
